# Beyond MRI: on the scientific value of combining non-human primate neuroimaging with metadata

**DOI:** 10.1016/j.neuroimage.2020.117679

**Published:** 2021-03

**Authors:** Colline Poirier, Suliann Ben Hamed, Pamela Garcia-Saldivar, Sze Chai Kwok, Adrien Meguerditchian, Hugo Merchant, Jeffrey Rogers, Sara Wells, Andrew S. Fox

**Affiliations:** a Biosciences Institute & Centre for Behaviour and Evolution, Faculty of Medical Sciences, Newcastle 6, UK; b Institut des Sciences Cognitives Marc Jeannerod, UMR 5229, Université de Lyon – CNRS, France; c Instituto de Neurobiología, UNAM, Campus Juriquilla. Boulevard Juriquilla No. 3001 Querétaro, Qro. 76230 México; d Shanghai Key Laboratory of Brain Functional Genomics, Key Laboratory of Brain Functional Genomics Ministry of Education, Shanghai Key Laboratory of Magnetic Resonance, Affiliated Mental Health Center (ECNU), Shanghai Changning Mental Health Center, School of Psychology and Cognitive Science, East China Normal University, Shanghai, China; e Division of Natural and Applied Sciences, Duke Kunshan University, Duke Institute for Brain Sciences, Kunshan, Jiangsu, China; f NYU-ECNU Institute of Brain and Cognitive Science at NYU Shanghai, Shanghai, China; g Laboratoire de Psychologie Cognitive, UMR7290, Université Aix-Marseille/CNRS, Institut Language, Communication and the Brain 13331 Marseille, France; h Human Genome Sequencing Center and Dept. of Molecular and Human Genetics, Baylor College of Medicine, Houston, Texas, USA 77030; i Centre for Macaques, MRC Harwell Institute, Porton Down, Salisbury, United Kingdom; j California National Primate Research Center, Department of Psychology, University of California, Davis, Davis, CA, 95616, USA

**Keywords:** Magnetic resonance imaging, Behaviour, Genetics, Physiology, Phylogenetics, BIDS

## Abstract

Sharing and pooling large amounts of non-human primate neuroimaging data offer new exciting opportunities to understand the primate brain. The potential of big data in non-human primate neuroimaging could however be tremendously enhanced by combining such neuroimaging data with other types of information. Here we describe metadata that have been identified as particularly valuable by the non-human primate neuroimaging community, including behavioural, genetic, physiological and phylogenetic data.

## Highlights

• Data sharing of primate neuroimaging offers new opportunities.
• The potential of metadata to enrich primate neuroimaging is described.
• Illustration of how meta-data can be shared in the BIDS format is provided.

## 1 Introduction

Non-human primate (NHP) neuroimaging offers unique opportunities to understand the primate brain (Phillips et al., 2014; Roeselfsema and Treue, 2014). However, this research field has been traditionally characterized by a single-laboratory approach where studies involve a small number of subjects (classically 2 to 3). Recently, the NHP neuroimaging community has come together to define its ambitions, based on a collaborative culture, and has embarked on a data sharing mission (Milham et al., 2018, 2020). International collaboration and data sharing open new avenues of research that could not be investigated at an individual lab scale, such as understanding inter-individual differences in brain structure and function, and the evolution of the primate brain. However, to take full advantage of its potential, big NHP neuroimaging data need to be enriched by accompanying information on the subjects (or species) who have undergone brain imaging, i.e. metadata. Here we describe metadata that have been identified as particularly promising. This list is not exhaustive and is likely to be expanded in the future. We finish by proposing how such metadata could be shared in a standardized way using the existing Brain Imaging Data Structure (BIDS) format.

## 2 Behavioural phenotyping data

For many researchers, the ultimate goal of neuroscience is to understand how the brain controls and is modified by behaviour. Taking advantage of within-species inter-individual variability, the combination of large-scale NHP behavioural phenotyping with neuroimaging data offers the possibility to investigate the neural substrates of primate behaviour. Due to the difficulty of scanning NHP subjects while they are awake, and the requirement that subjects remain still during the scanning session, task-related fMRI in NHP is rare and most studies have focused on perceptual tasks in two or three subjects (e.g. Poirier et al., 2017; Toarmino et al., 2017; Clery et al., 2018). To understand inter-subject variability, the most promising approach is to correlate inter-individual differences in brain structure and function at rest with behaviour measured outside the scanner. Two types of behavioural data can be collected off-line: stimulus-induced behaviour and spontaneous home-cage behaviour. The standardization of stimulus-induced tests (e.g. cognitive tasks, temperament tests), combined with data sharing, offer the opportunity to collect both behavioural and MRI data in a large number of subjects (e.g. Oler et al., 2010; Birn et al., 2014; Fox et al., 2015). The generalization of automatic testing within the home-cage where individuals interact willingly with a protected computing interface (Calapai et al., 2017;Fizet et al., 2017; Butler and Kennedy, 2019) should facilitate the collection of such data. Task-related behaviour however only gives us a restricted view on NHP behaviour and cognition, the subject's behaviour being constrained by the task designed by humans. Another complementary approach is to link neural inter-individual variability with spontaneous, natural behaviour, displayed by individuals in their home cage. This approach is particularly promising when individuals are housed with conspecifics, allowing them to display a comprehensive range of natural behaviours, including social ones. This approach is currently hampered by a lack of technology to automatically measure spontaneous behaviour in large numbers of animals. Several attempts to automatically analyse video recordings of NHP spontaneous behaviour (in their cage or in a natural environment) using artificial intelligence are currently on-going. Individual subjects can now be identified in complex social settings (Witham, 2018; Shukla et al., 2019). Pose estimator tools, which allow tracking several parts of an animal's body in relation to each other and to the physical environment, have recently been developed and applied to macaques (Labuguen et al., 2019; Bala, 2020). However, what is currently missing is a tool able to automatically interpret this kinematic information into complex, ethologically relevant behaviours. When this hurdle is overcome, and the algorithm shared with the international community, providing standardized data of home-cage behaviour along with individual MRI scans will be straightforward.

## 3 Genotyping data

Neuroimaging data, both structural and functional, provide insight into the correlations between expressed behaviour and the neural circuits that drive and execute particular behaviours. However, a more complete understanding of the underlying cellular biology can be achieved if we investigate the relationships between genes, brains, and behaviour. This approach will allow researchers to investigate the genetic mechanisms that shape brain development, contribute to juvenile or adult neurophysiology, and ultimately influence behaviour.

It is well established that environmental exposure and developmental experience, especially early in life, can influence neurodevelopment and thus affect juvenile and adult behaviour. Adverse developmental environments such as nursery rearing with lack of a maternal relationship, as opposed to normal maternal rearing, can alter behavioural outcomes (Bastian et al., 2003; Higley et al., 1991). While acknowledging the potential effects of early rearing and developmental conditions on NHP neurobiology and behaviour, it is also critical to consider the effects of inherited genetic variation. The evidence supporting strong genetic effects on both risk for psychiatric illness and individual variation in normal behaviour among humans is substantial and definitive (Cloninger et al., 2019; Rees & Owen, 2020). Though fewer genetic analyses have been performed in NHP, it is clear that in NHP as well as in humans, genetic variation among individuals of the same species does influence variation in both neural circuit function (Fears et al., 2009; Oler et al., 2010) and expressed behaviour (Hopkins et al., 2014b; Johnson et al., 2015; Rogers, 2018, Rogers et al., 2013a). Therefore, in order to fully understand the causes of behavioural variation, and to identify the factors that drive individual differences in brain structure and function, it is important to investigate the influence of genetic variation on these outcome phenotypes.

Genetic analyses of neuroimaging or behavioural data can take several forms. One fundamental approach is to use the tools of quantitative genetics to estimate the additive genetic heritability of specific phenotypes measured either through neuroimaging or behavioural observations. Analyses of additive genetic heritability provide quantitative estimates of the proportion of total phenotypic variation in a given study population that is attributable to genetic differences among those individuals (Falconer, 1997). Heritability can be estimated in various ways, but among the most common are analyses of phenotypic variation across either a population of inter-related individuals with known pedigree relationships or across sets of fraternal and identical twins. Both approaches exploit the expectation that if a particular phenotype is influenced by genetic differences among individuals, then the pair-wise phenotypic differences across all pairs in a population should be less for pairs that share more genes through common descent (i.e. have higher pairwise kinship values) than for pairs that are more distantly related and thus share a lower proportion of genes in common. Various studies have reported significant heritability for a variety of NHP behaviours (Fairbanks et al., 2004; Fawcett et al., 2014; Hopkins, et al., 2014a) as well as for neuroimaging phenotypes (Lyons et al., 2001; Rogers et al., 2010; Oler et al., 2010; Fox et al., 2015; Fox et al., 2018; Tromp et al., 2019).

The second fundamental approach to genetic analysis is genetic association testing to identify the specific genes and polymorphisms that contribute to brain function and behaviour. In this case, specific genotypes within known genes or other DNA sequences are scored across a set of individuals in a population and a statistical test for association between genotype and phenotype is performed. These studies often use large cohorts of unrelated individuals, which avoids undetected genetic correlations among individuals that can skew test statistics. However, increased statistical power can be obtained by performing genetic association in cohorts that include individuals with close or moderate kinship relationships (Blangero et al., 2001; Lippert et al., 2011; Glahn et al., 2019). In these circumstances, it is necessary to account for relatedness when testing for significant association between specific genotypes and a given phenotype of interest.

NHPs provide a powerful opportunity for identifying genes that contribute to complex phenotypes. In particular, compared to modern humans, who underwent an evolutionary population bottleneck, NHP have been shown to have greater levels of within-species DNA sequence variation, lower linkage disequilibrium and a larger number of predicted deleterious polymorphisms (Gibbs et al., 2007. Rogers et al., 2006; Xue et al., 2016; Bimber et al., 2017). The evidence for both heritability and particular genetic associations affecting behaviour or neuroimaging data in NHP is growing, reinforcing the utility of NHP models (Rogers, 2018). There is now a significant opportunity to advance our understanding of the factors that influence individual variation by incorporating genetic tests into future projects. Increasingly, research colonies of NHP identify and make available the kinship relationships among their research animals, and this makes quantitative genetic analyses of heritability straightforward. Documenting the heritability of complex traits such as neuroanatomy or neural circuit activity provides the basis for subsequent studies intended to identify the genes involved, or on focused exploration of the interactions between genetic variation and environment influences on development. The cost of large-scale genotyping, whole genome or whole exome sequencing of NHP is consistently decreasing. This makes large-scale genome-wide genetic association studies more feasible than they have been in the past. Overall, the prospects for incorporating genetic analyses into research programs investigating neuroanatomical structure, neural circuit function or expressed behaviour are improving rapidly. This includes increasing opportunity to understand differential gene expression across individuals within a species or between species using RNA sequencing. We recommend that researchers store tissue samples (e.g. blood) or extracted DNA from animals that undergo MRI scans, even if they have no immediate plans for sequencing. National breeding centres might help storing and/or processing these samples, if necessary. DNA samples will facilitate future large-scale efforts to aggregate primate data to identify the genetic contributions to brain structure and function. The scientific benefits of this will be tremendous as such studies provide the basis for a more complete and detailed understanding of the biological factors that influence NHP neurobiology and behaviour, and facilitate integrative cross-species genomic analyses.

## 4 Welfare information

To understand environmental contributions to brain structure and function, researchers must consider the animals past and present welfare. One way to assess NHP welfare is to rely on physical and social characteristics of their past and present environment. Rearing history (mother/peer-rearing; rearing cage size; weaning age), social nature of current housing (single-, pair- or social housing), cage size, presence of foraging opportunity, and other types of physical enrichment should be easy to collect and share along each MRI dataset. These kinds of data are already beginning to provide insight, as rearing history, cage size, and number of cagemates have been shown to impact neuroimaging measures (e.g. Sallet et al., 2011; Noonan et al., 2014; Howell et al., 2019). However, these environmental characteristics only provide indirect evidence about NHP welfare and do not take into account the fact that different animals may react differently to a similar environment. Another way to assess NHP welfare is to take individual-based measures. Body weight and body condition can be measured in a standardised way but probably lack sensitivity to detect anything but the biggest welfare problems. In-depth welfare assessment requires incorporating regular behavioural observations of each individual. While such direct observations are usually made in every primate facility, there are usually not done in a standardised way. Such a standardization relies on using the same list of quantifiable behaviours, the same behavioural definitions and the same data collection protocols. While standardization is already challenging to obtain within one facility, it is even more difficult to achieve across facilities. In addition, the mere presence of a human observer at the cage side tends to modify macaques’ behaviour, leading to inaccurate quantification of behavioural welfare indicators (Iredale et al., 2010). The gold standard in behavioural welfare assessment consists of systematic quantification of individuals’ behaviour via remotely-controlled cameras. However, considering the human resource necessary to analyse manually these videos, it is currently not feasible to collect and share such detailed behavioural data for large numbers of individuals. The automatic identification and quantification of NHP behaviours from video-recordings will allow achieving this goal in the future.

Independently of their nature, welfare assessment measures can be used for different purposes, when shared along with individual MRI scans. The variability in MRI data associated with different welfare status could be treated either as noise or as a source of invaluable information. In the first case, welfare assessment measures can be used to harmonize MRI samples, by controlling statistically for the variability associated with welfare measures. Alternatively, the impact of diverse welfare status on brain structure and function might be the object of scientific investigation as they might shed light on the impact of similarly diverse levels of well-being in the human population. Even in studies whose primary topic of investigation is not the impact of welfare on brain structure and function, the sharing of large amounts of data from NHPs of different welfare status offers the opportunity to investigate the interaction between the primary factor of interest (e.g. ageing, cognition, genetics) and welfare. Such studies are likely to be more representative of the large variability in well-being observed in human beings. From an ethical point of view, while many (including the present authors) consider improving the welfare status of experimental NHP over the world as a crucial goal, it might be argued that researchers have the moral duty, in the meantime, to make use of this NHP welfare variability to maximise the amount of knowledge provided by NHP experiments.

## 5 Manual lateralization

A prominent feature of the human brain is its hemispheric specialization, which refers to the functional lateralization of the brain for a particular cognitive process, as well as to interhemispheric anatomical asymmetries for specific structures. In humans, handedness is one the best-known behavioural manifestations of such a hemispheric specialization including the primary motor cortex along the central sulcus (Hammond, 2002; Amunts et al., 1996; 2000; Foundas et al., 1998). About 90% of humans are right-handed regardless of cultures (Annett, 1985; Marchant, McGrew & Eibl-Eibesfeldt, 1995). Left-handedness has historically been considered as different in terms of hemispheric organization including lateralization of language processing or spatial cognition, compared to right- handedness (Tzourio et al., 1998; Zago et al., 2016; O'Regan & Serrien, 2018), although recent findings report that a large majority of left-handed individual showed also left-hemispheric specialization for language processing, just like right-handed people (Knecht et al, 2000; Mazoyer et al., 2014). As a result, given that handedness of participants might represent a potential critical source of variability in the brain (see also Bryden, 1987), most neuropsychological studies limit such brain variability across participants by either including sample size carefully balanced for handedness or targeting exclusively right-handed participants.

In contrast, neuroimaging studies conducted in NHP have never considered controlling the handedness of subjects when developing their sample population, although such a question should be debated in the community. One of the potential explanations is that reports of manual laterality in NHP were historically considered as inconsistent across the literature if not non-existent, suggesting the human's uniqueness for both predominance of right-handedness and hemispheric specialization (Warren, 1980; McGrew and Marchant, 1997; Crow, 2004; Cashmore et al., 2008).

However, this view has been challenged by a large body of recent evidence showing brain and behavioural asymmetries in many vertebrates including NHP (Marie et al. 2018; Rogers et al., 2013b). To address the inconsistency of the primate handedness literature, some authors pointed out that most of these studies focused on simple unimanual behaviours (e.g., objects reaching) for quantifying hand preference (reviewed in Papademetriou, Sheu & Michel, 2005). In fact, in contrast to more complex manual tasks, simple unimanual behaviours turned out to be poor measures of handedness given (1) their sensitiveness to biases related to confounding situational factors such as the subjects’ posture or the initial position of object to reach and (2) their related low motor demands which do not especially require the use of the preferred hand. As a result, simple unimanual behaviours were then considered as inappropriate for detecting individual hand preference (Meguerditchian et al., 2013).

It is now well acknowledged that the complexity of manual tasks is a critical factor to consider for assessing hand preference (Fagot & Vauclair, 1991; Meguerditchian et al., 2013). There are an increasing number of handedness studies in NHP which reported robust individual hand preference for complex manual tasks such as tool use (Lonsdorf and Hopkins, 2005; Hopkins et al., 2009), bimanual coordinated action (reviewed in Meguerditchian et al., 2013), and gestures (Meguerditchian & Vauclair, 2006; Meguerditchian et al., 2011; Prieur et al., 2017). The bimanual coordinated tube task (video 1), initially designed by William Hopkins for testing chimpanzees (Hopkins, 1995), has received special attention for assessing hand preferences in NHP for the following reasons. First, the tube task, which consists of holding an opaque PVC tube with one hand and removing food inside the tube with the fingers of the other “dominant” hand, is easy to propose to a primate individual and to be performed by any primate species (in contrast to tool use). This has favoured its generalization for a large comparative perspective on primate handedness. Second, the type of bimanual coordination induced by the tube task allows minimizing confounding situational factors by standardizing subject posture as well as the position of the tube for retrieving food across data points within a subject, across subjects and across species. Third, it is complex enough to elicit high motor demands that result in robust and consistent individual hand preference across time (e.g. Molesti et al., 2016). Fourth, the bimanual coordinated tube task was found to elicit robust population-level right-handedness mostly in terrestrial NHP including baboons, gorillas, chimpanzees and humans, and population-level left-handedness in arboreal species such as squirrel monkeys, orangutans and snub-nosed monkeys (Meguerditchian et al., 2013). Regarding the two main primate species used in MRI neuroimaging – macaques and marmosets – the existence of a population-level handedness remains unclear for bimanual coordinated tasks. In macaque species, strong individual hand preferences have been reported, but direction of population-level handedness seem to differ across studies (Meguerditchian et al., 2013 for a review). In marmosets, to our knowledge, no data related to bimanual tasks are available so far. One reason is that New World monkeys such as marmosets or squirrel monkeys are not able to insert a single finger inside the tube to remove the food. So, the tube task must be adapted in order to allow these monkeys to insert the whole hand inside the tube as this has been done in a study in squirrel monkeys (see Meguerditchian et al., 2012). Finally, direction and degree of hand preference for the tube task in NHP such as baboons, capuchin monkeys, squirrel monkeys or chimpanzees have been found to be associated with contralateral neuro-structural asymmetries in the primary motor cortex including the surface of the motor hand area surface, its neuronal densities or its adjacent Central sulcus depth (Nudo et al., 1992; Hopkins and Cantalupo, 2004; Dadda et al., 2006; Sherwood et al., 2007; Phillips & Sherwood, 2005; Hopkins, 2013; Margiotoudi et al., 2019). These latter studies clearly suggest that, just like in humans, handedness in NHP is a robust lateralization phenomenon which reflects hemispheric specialization of the brain. In humans, handedness and the associated motor cortex lateralization often co-vary with more complex lateralization patterns in the parietal, temporal, prefrontal and frontal cortex (e.g. Fischer et al., 1991; Frässle et al., 2016; Morita et al., 2020). In NHP, while a lateralisation of neural mechanisms underlying cognitive processes have been anecdotally reported, a systematic evaluation of this fundamental neuroscience question is still missing. A systematic documentation of handedness when collecting NHP MRI data may thus serve to investigate whether individual handedness and motor cortex lateralization also co-vary with a lateralization of other brain organisational features (e.g. resting-state networks, or other localizer-based functional regions of interest, see Russ, Ben Hamed et al., this issue).

In conclusion, we believe there is now enough evidence to speak (1) for a primate continuity with human handedness and thus (2) for the generalization of the use of bimanual coordinated behaviours - such as the tube task - for controlling or assessing the impact of individual handedness on brain structure and function.

## 6 Fluid control

**Fig. 1:**
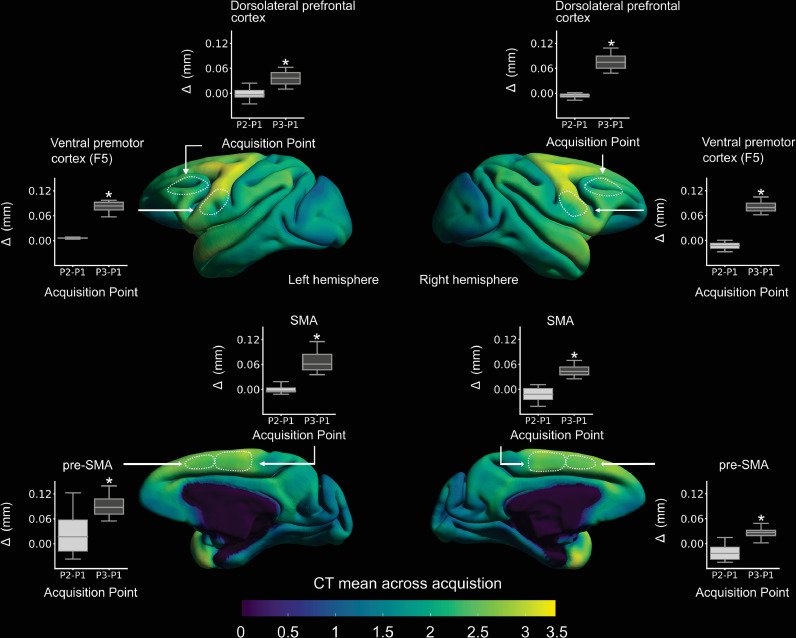
Scan-rescan variability in cortical thickness during free water and water control conditions in one Rhesus macaque. Scans P1 and P2 were both acquired when the subject had unlimited access to water for at least one month, and were separated by 13 days. P3 was acquired at the end of a 24-day period of fluid control that started immediately after P2. The fluid control protocol consisted in access to 70% of free intake, 6 days a week. Regions-of-interest were chosen randomly. CT. Cortical Thickness. SMA. Supplementary Motor Area. pre-SMA. Pre-Supplementary Motor Area.*paired-t-test, *p* < 0.0001. Note that the difference is of small amplitude but consistent across brain regions. These results come from one subject and need to be replicated.

During neuroimaging experiments in awake NHPs, animals are often rewarded by a drop of fluid for staying motionless and/or for successfully performing the requested task. When fluid preference has been measured at the individual level and the subject-specific preferred fluid is used (Gray et al., 2019), this approach can be sufficient to acquire high quality data. However, when a high number of trials is necessary to obtain statistically robust results, it is sometimes necessary to combine this strategy with some level of fluid control or restriction outside the scanner. The severity of fluid control varies greatly between sites, not only in terms of amounts of fluid accessible by each individual but also in terms of timing (when the animal has access to fluids). A minimal amount of fluid is always provided to each animal but this amount can be measured based on the animal's weight or based on its consumption when it has free access to fluids. When an animal does not perform well enough in the scanner to gain access to this minimal amount of fluid, the remaining amount can be given at the end of the day or at the end of the week. There is also variation in the maximal amount of fluid accessible to each NHP. In some laboratories, animals are allowed to drink as much as they want in the scanner as long as they perform well behaviourally. In other places, the maximal amount per day is capped to ensure that animals are still motivated the following day. The fluid restriction protocol can also be applied 7 days a week or be relaxed during week-ends (when animals have free access to water or access to a quantity similar to what they usually drink outside of fluid control periods). Despite the fact that every protocol ensures that the long-term physiology of the animals is not compromised, the variation in fluid restriction protocols induces a variation in the hydration level of NHPs when they are scanned, which can result in contrast differences in MRI images. Such differences might affect the estimation of classical measures such as BOLD signal, cortical thickness, and voxel-based morphometry metrics (fig. 1). When conjointly analysing MRI datasets from different sites, researchers might therefore be interested in controlling this source of variability. Another source of variability in MRI datasets is the potential impact of fluid control on primate psychological well-being. This impact is likely to depend on the details of the fluid control protocols. Researchers usually ensure that this impact is not too severe by ensuring that NHP subjects maintain a healthy body weight. However, more subtle consequences cannot be excluded without a systematic assessment of subjects’ well-being using more sensitive measures (e.g. Hage et al., 2014; Gray et al. 2016; Pfefferle et al., 2018). Such assessments at the behavioural level are difficult (see section 4) and suffer from methodological limitations (e.g. Poirier and Bateson, 2017; Poirier et al., 2019a). Large MRI datasets where variation in fluid restriction is not confounded with scanning sites offer the unique opportunity to investigate the consequences of fluid control on subjects’ well-being at the neural level (Poirier et al., 2019b), and, if metadata related to spontaneous home cage behaviour are also shared, at the behavioural level as well. Such data would allow researchers to scientifically assess and if necessary refine fluid control protocols, in line with the 3R's (Russell and Burch, 1959).

## 7 Metadata specific to functional MRI

Functional MRI relies on an indirect measure of neuronal activity. This information is corrupted by multiple sources of noise (Caballero-Gaudes and Reynolds, 2017). Part of this noise is of instrumental origin (e.g. instrumental drifts, hardware instabilities, signal changes due to head motion etc.). Another contributor is of physiological non-neuronal origin (e.g. cardiac and respiratory noise, changes in arterial CO2 or blood pressure, vasomotion, cerebral autoregulation mechanisms).

A third part is of neuronal origin (e.g. eye movements, arousal, blinks). Signal denoising is thus a crucial part of data analysis pipelines, both in task-based and in resting state fMRI studies (Caballero-Gaudes and Reynolds, 2017; Murphy et al., 2013). Some of these denoising approaches are data-driven, based on data decomposition methods such as principal or independent component analysis. These are blind to actual sources of noise and are extremely useful in cases in which physiological monitoring is unavailable. Other signal denoising approaches are based on metadata recorded synchronously with the fMRI data. These metadata are realigned to fMRI time series and down sampled at a repetition time (TR) resolution to generate nuisance regressors. These can then be introduced in a regression model in order to account for their explained variance. The use of such regressors of non-interest must be balanced against the loss of degrees of freedom since it is not possible to know whether all noise related effects in the signal are being removed, or whether neuronal-related fluctuations are also being removed.

A first obvious source of non-neuronal variability that is classically taken into account is motion artefacts, by adding, after volume registration, the time series of the six estimated translational and rotational realignment parameters as nuisance regressors in the regression model. While all processing pipelines involve motion artefact correction, it is worth noting that new multi-shot 3D EPI acquisitions are more sensitive to physiological noise in general and to motion artefacts in particular, than single-shot 2D EPI acquisitions. In addition, it is important to keep in mind that awake NHP experiments, in which animals are typically head-fixed, involve specific motion artefacts that cannot always be minimized by training, such as swallowing, task-related head movements or specific reward intake movements.

A second source of (mostly) non-neuronal variability that is highly relevant to both anesthetized and awake NHP fMRI is heart and respiratory rate. Amongst other effects (see for exhaustive description, Caballero-Gaudes and Reynolds, 2018), blood pulsation induces small changes in brain tissue close to blood vessels (e.g. around the sagittal sinus, the edges of the brain and in the sulci). Blood pulsation and respiration also result in motion of large brain regions such as the thalamus, the diencephalon or the brain stem. In the anesthetized monkey, this information can be recorded using pulse oximetry (measuring changes in the infrared light absorbed by blood infused tissue, due to global changes in oxygenation levels) or electrocardiograms (ECG). Respiration rate can be recorded directly from the ventilator that is maintaining anaesthesia. Alternatively, if respiration signals are unavailable, they can also be extracted from the ECG signals (Labate et al., 2013). Heart and respiratory rate are highly dependent on anaesthesia and medication kinetics and may vary during data acquisition if care is not taken to prevent this. It is typically easier to stabilize these parameters using gas or perfusion anaesthesia. In awake monkey fMRI experiments, although heart and respiratory rates are challenging to record, they are important to track. Indeed, heart and respiratory rates are typically non-stationary and can thus not be removed by a simple band-pass filter. Crucially, heart rate variability is considered as a marker of the activity of the autonomous system (Berntson et al., 1997). In humans, this variability is associated with fluctuations in resting-state functional connectivity between cortical regions involved in vigilance and arousal (Chang et al., 2013). Human heart rate variability has also been shown to correlate with local changes in BOLD signal in experiments involving emotions (Critchley et al., 2005), pain (Sclocco et al., 2016), cognition (Basile et al., 2013), autonomic nervous system modulation (Napadow et al., 2008). As is the case in humans, the emotional reaction to passively viewed visual stimuli induces heart rate variations in macaques (Bliss-Moreau et al., 2013). Heart rate variability in NHPs is thus expected to follow the same modulators as evidenced in humans, and to modulate NHP hemodynamic responses in the same way. New methods allow for heart rate estimation from high resolution NHP facial video images (Unakafov et al., 2018), including during scanning of awake NHPs viewing emotional stimuli (Froesel et al., 2020).

In awake NHP imaging, another source of noise in the fMRI data important to consider is eye-related data. Acquiring this metadata requires an MRI compatible eye tracker, as well as a minimal training in order to calibrate eye information. In minimally trained animals, coarse eye calibration can be achieved by having monkeys gaze at information rich small images. The size of the image will determine the precision of the calibration. In highly trained animals, eye tracking calibration involves fixating stimuli of only a few pixels in size and discriminating a colour change for a reward. Eye tracking information is very rich and multiple distinct signals can be extracted. Epochs of eyes open vs. eyes closed, that have been associated with changes in global neuronal signal amplitude, can for example be estimated (Wong et al., 2015). More specific information on eye position and saccades, blinks and pupil size can also be extracted, all of which have been associated with specific cortical activations. As a result, not considering them as regressors of non-interest can lead to important confounds both in resting state experiments (reporting temporal correlations biased by these eye signals) as well as during task-based experiments, when eye signals correlate with task timings (and are thus not averaged out during data analysis). For example, spontaneous blinks are mostly generated to lubricate the cornea when needed. They have consequences on the brain not only in the visual cortex (Hupé et al., 2012), but also in cortical regions processing the somatosensory, proprioceptive, peripheral visual, and possibly nociceptive consequences of blinks (Guipponi et al., 2015, Cléry et al., 2018). This can result in the contamination of fMRI protocols that generate heterogeneous blink behaviours. In particular, during active cognitive behaviour, spontaneous blinks are suggested to be actively involved in attention disengagement, coinciding with a deactivation of the dorsal attentional network as well as an activation of the default-mode network (Nakano et al., 2013).

Tracking eye position is behaviourally informative, both during resting-state scans in which monkeys are expected not to fall asleep, during the viewing of information rich naturalistic movies as well as during more complex cognitive tasks. In the absence of MRI compatible eye tracking technology, machine learning can be used to estimate the direction of gaze from the fMRI time series of the eye voxels at a TR resolution. This approach has been developed in humans (Son et al., 2019, LaConte et al., 2006, 2007) and is being tested in NHP experiments (Russ et al., personal communication). When eye position is used as a signal regressor, the parieto-frontal oculomotor network is typically identified, as well as premotor areas (Koyama et al., 2004) and cingulate face areas (Cléry et al., 2018). Using eye position as a nuisance regressor is thus important not to confound reported observations (whether in terms of inter-areal correlations or in terms of % of signal change) by this variable, which has been repeatedly reported to be a strong behavioural marker of overt cognitive processes.

Both spontaneous blink and eye movement frequency can be taken as an index of the level of the monkey's vigilance and engagement in the task, whether during unrestrained resting-state scans, during resting state scans imposing fixation or during more complex tasks. In this respect, pupil data can also be extremely useful to objectify such variations in states of vigilance (Pais-Roldan et al., 2020). Pupil diameter is known to be associated with Locus Coeruleus noradrenergic neuronal firing. Pupil size positively correlates with BOLD activations in the Locus Coeruleus, thalamus, posterior cingulate cortex, dorsal anterior cingulate and paracingulate cortex, orbitofrontal cortex, and right anterior insular cortex, i.e. in brain regions associated with selective attention, salience, error-detection and decision-making (Yellin et al., 2015, DiNuzzo et al., 2019).

Overall, the systematic recording of the above discussed metadata would allow a better denoising of fMRI data. In addition, the availability of these metadata on a larger set of animals would facilitate a better assessment of the neuronal processes associated with each of these physiological and behavioural measures. Last, this would allow the investigation of individual differences in cognitive processes indexed by some of these metadata.

## 8 Phylogenetic information

Understanding primate cognition from an ethological perspective requires us to study its mechanisms, ontogeny (development), function, and phylogeny (evolution) (Tinbergen, 1963). A popular approach to study the evolution of cognition consists of comparing cognitive abilities of different primate species and their underlying brain structure and function. So far, comparative studies of primate cognition have been dominated by behavioural assays, with few studies using a neuroscience approach (Balezeau et al., 2020; Duong, 2010; Rilling, 2014). Between-species behavioural and brain variability has also mainly been assessed from a qualitative point of view (for exceptions, see for instance Barton 1998; Dunbar 1998b; Deaner et al. 2000, 2007; Lindenfors et al. 2007; Isler and van Schaik 2009).

Phylogenetics is the study of the evolutionary history and relationships among individuals or groups of organisms (e.g. species or populations). Traditionally, the relationships between (sub-) species are analysed and understood via phylogenetic methods that evaluate observed heritable traits, such as DNA sequences or morphology under a model of evolution of these traits. The result of these analyses is a phylogeny (also known as a phylogenetic tree) – a diagrammatic display and hypothesis about the history of the evolutionary relationships of a group of organisms (Felsenstein, 1985). Given that species with more shared ancestry are expected to perform cognitive tasks in a more similar way than more distantly related species (MacLean et al., 2012), phylogenetics can also be applied to cognition. This approach offers the opportunity to assess quantitative differences between species, taking into account shared ancestry, and to infer the evolutionary history of cognitive traits. However, it requires extensive datasets covering a large variety of species.

The field of primate cognition has recently seen the emergence of an international consortium to support large-scale collaborations in primate behavioural research (Altschul and Beran, 2019a, Altschul and Beran, 2019b). This initiative has produced one of the largest and most diverse primate datasets describing behaviour, enabling researchers to address novel research questions regarding the evolution of primate cognition (in this case, short-term memory).

The recent expansion of neuroimaging to several NHP species, combined with large-scale data sharing initiatives (Milham et al., 2018, 2020), offer the opportunity to follow a similar approach. It is worth noting that current European (as of 2010) and US (as of 2015) regulations do not authorize experimental research in great apes (e.g. gorillas, orang-utans and chimpanzees). Thus, for these species, neuroimaging data are either collected post-mortem or during veterinary investigations related to the individual's health. Combining primate neuroimaging data from multiple species with primate phylogenetic trees (for a digital version, see Arnold et al., 2010) will allow investigating (Freckleton et al. 2002, MacLean and Hare, 2012) or controlling for (Grafen 1989; Pagel 1999) the variability in species differences due to shared ancestry. Examples of potential applications include quantifying how much of the between-species variability of any neuroimaging trait (e.g. cortical thickness, brain gyrification, connectivity profile of a specific brain region) is due to shared ancestry (phylogenetic signal) and how much is due to other factors (e.g. a shared physical environment or shared level of complexity in social relationships), and inferring this trait in common ancestors (Heuer et al., 2019). Ultimately, comparing and merging the evolutionary history of cognitive traits from a behavioural, neural, and genetic point of view promise to transform our understanding of primate cognition.

## 9 Organization of metadata sharing: Introduction to BIDS

Neuroimaging experiments are complicated and often require relevant scan parameters and associated metadata for interpretation. Data can be organized in many different ways, and a lack of standard organization can lead to misunderstandings when sharing data, even for similar experiments from the same lab. Recent efforts to develop consensus on how to organize neuroimaging data have led to the development of the BIDS (Gorgolewski, et al., 2016). BIDS is a consensus framework for how to organize neuroimaging data and associated metadata. The BIDS format makes it easy for other investigators to understand what data was collected to facilitate collaboration. In addition, BIDS-Apps (http://bids-apps.neuroimaging.io/) and other software tools can interpret BIDS formatted data, greatly simplifying the analysis process and providing an opportunity to standardize analysis pipelines. The open-science neuroimaging data-sharing resource PRIMatE Data Exchange (PRIME-DE) accepts and encourages contributors to upload BIDS formatted data. By sharing data in BIDS format, neuroimaging methods research can use the large-scale neuroimaging datasets, such as PRIME-DE, to further test, develop, and optimize NHP neuroimaging tools.

The full BIDS specification can be found at https://bids-specification.readthedocs.io/en/stable. In brief, it specifies a standard naming structure for reconstructed images and folders, which include multiple key-value pairs separated by a “_“ with keys and values separated by a “-“. For example, each functional scan is stored as a 4-D niftii named *sub-XX_task-XX_bold.nii.gz* file in *dataset/subj-XX/func*, and each T1-anatomical data is stored as a 3-D niftii file named sub-XX_T1w.nii.gz in *dataset/subj-XX/anat*. There are a number of tools that can help researchers get their data into BIDS format (e.g. HeuDiConv https://neuroimaging-core-docs.readthedocs.io/en/latest/pages/heudiconv.html) and validate that a BIDS dataset (e.g. BIDS-validator https://bids-standard.github.io/bids-validator/). These tools are rapidly improving, and evolving, to further simplify the process of converting DICOM data into this standardized BIDS format to facilitate data sharing and homogenized analyses. We realize that many existing, ongoing, and future projects will identify study-specific reasons to use their own data structure. To address this problem, researchers can write scripts to convert between various standardized data formats. In the context of the Human Connectome Project (HCP), for example, researchers have created heuristics for converting from HCP to BIDS, as well as BIDS-Apps that are designed to run HCP processing pipelines on BIDS datasets (https://github.com/BIDS-Apps/HCPPipelines; Glasser et al. 2013, Smith et al. 2013).

**Fig.2:**
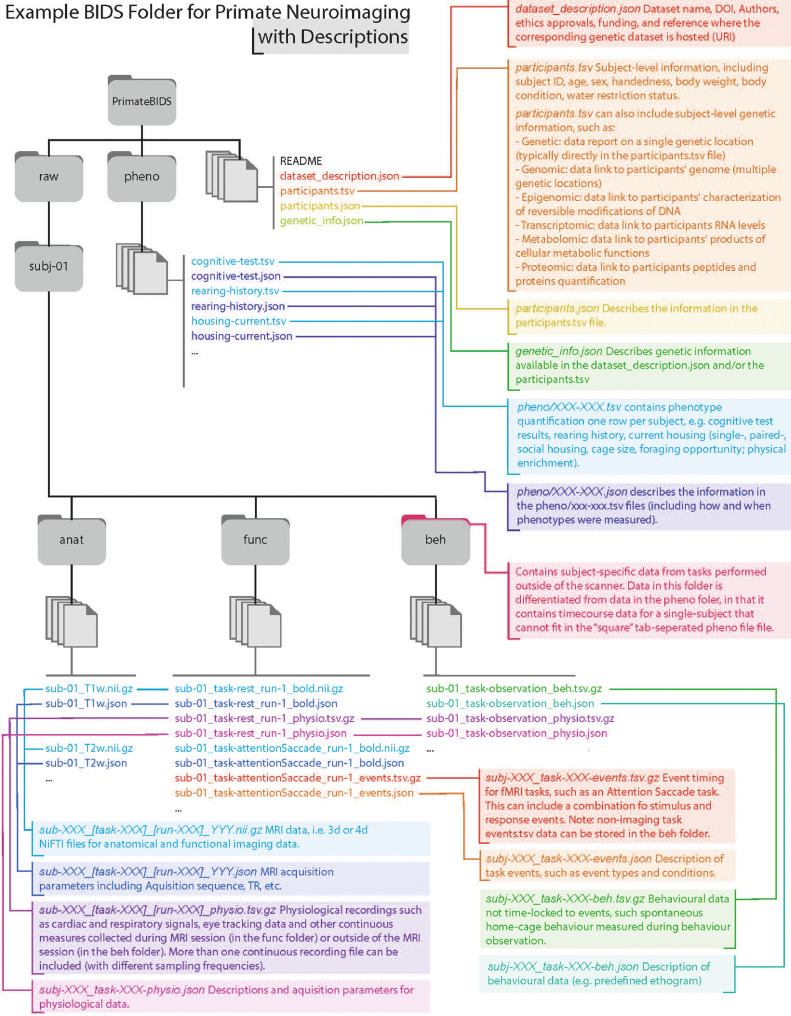
Example BIDS Folder for Primate Neuroimaging with Metadata. The BIDS format describes a unified structure for sharing brain imaging data in files and folders (grey). Here we show the organization of an example BIDS dataset, with file descriptions (connected to files with coloured lines). Briefly, each study has a top-level directory (e.g. *PrimateBIDS*), which contains folders for raw data (*raw*) and phenotype information (*pheno*), along with descriptor files where researchers can share descriptions of the subjects, dataset, and accompanying genetic information. The *pheno* folder houses across-subject data (*.tsv*) and descriptions of those data (*.json*). The *raw* folder contains a folder for each subject, e.g. *subj-XX*. Each subject-folder, in turn, contains *anat, func*, and *beh* folders, which contain anatomical, functional, and behavioural data, respectively. Similar to the pheno folder, each of these folders contains data (e.g. *.nii.gz* or *.tsv*) with an accompanying descriptor file (*.json*), which describes relevant acquisition/quantification parameters. Individual subject folders can be used to share any data that cannot be easily summarized into an across-subject tabular *.tsv* file in the *pheno* folder.

In addition to neuroimaging data, the BIDS format has been expanded to allow for standardized sharing of related metadata when available, including in-scanner task/manipulation data (including different “resting” conditions, such as levels of anaesthesia), in-scanner physiological data, as well as behavioural data collected outside of the scanner, rearing information (e.g. early-life rearing/housing information), and genetic descriptors (including single-nucleotide polymorphisms and whole-genome sequences). Fig. 2 outlines how to incorporate the data described above into the BIDS format.

Some types of data, including genetic information, can be shared in multiple ways. Individual SNP data can be shared in the tabular dataset/participants.tsv file, with columns for individual SNP locations and rows for subjects. If whole-genome sequencing data is available, information about, and links to, this data can be shared in the dataset/genetic_info.json file. Importantly, the sharing of genetic information is not limited to DNA, but can include epigenetic, transcriptomic, metabolomic, and proteomic data (see Fig 2). As with genomic data, these data can be shared for individual sites in the dataset/participants.tsv or link to larger datasets in the dataset/genetic_info.json file. Additional details can be found in the Genetic Descriptor BIDS specification (https://bids-specification.readthedocs.io/en/latest/04-modality-specific-files/08-genetic-descriptor.html).

Behavioural data acquired outside the MRI scanner can also be shared in different places, at the study level, in the *dataset/pheno/* folder, and at the subject level, in the *dataset/subj-XX/beh/* folder. The *pheno* folder contains *.tsv* files with tabular measure by subject data, i.e. one subject per row, and one column per measure. Each *.tsv* file is accompanied by a .*json* file, which describes the measures for which there are data. The *pheno* folder is intended for storing phenotypic data that is easily summarized at the subject level. For instance, summary statistics from standardized cognitive tests should be shared here. For more complex data, BIDS also specifies a *dataset/subj-XXX/beh/* folder, within each subject directory. The *Beh* folder contains .*tsv* files where rows denote time/trial rather than subject, and accompanying .*json* files that clearly describe the contents of the .*tsv* files. Depending on whether behavioural data are time-locked to events or not, they can be stored in .tsv files named *subj-XXX_task-XXX-events.tsv.gz* or *subj-XXX_task-XXX-beh.tsv.gz*, respectively (Fig. 2). The *Beh* folder is designed to contain experimental or observational data that cannot be easily summarized into a tabular format across-subjects, or that would benefit from being shared in their raw format to allow researchers to derive multiple phenotypic measures. Examples of such data include non-standardised cognitive tests and observations of home cage spontaneous behaviour. As this can be confusing, it is worth reiterating that BIDS is designed to store behavioural data in two locations, the *dataset/pheno/* folder, for tabular across-subjects data, and the *dataset/subj-XX/beh/* folder, for individual subject data that cannot be easily coerced into a across-subjects tabular format.

## 10 Conclusion

Combined with open sharing of neuroimaging data, metadata has the power to transform primate neuroimaging. Metadata will help removing noise from the datasets, increasing the statistical power of studies that combine datasets from different facilities. Perhaps more importantly, they will also allow the integration of neuroimaging data into a multi-disciplinary framework, providing an integrative view of the primate brain within a wider context of primate biology, to understand its mechanisms and evolution.

Here, we provide suggestions for what metadata to share, along with an introduction to the BIDS framework which facilitates sharing these data in a standard form. BIDS allows researchers to share behavioural data (e.g. task performance, home cage spontaneous behaviours), welfare data (e.g. rearing history, housing), genetic data (e.g whole-genome sequencing), manual lateralization data (i.e. estimated handedness), fluid information (i.e. fluid intake and control), physiological data (e.g. ECG), acquisition conditions (e.g. awake-task, anaesthesia levels), as well as neuroimaging data (e.g. BOLD, diffusion-imaging) and relevant descriptors (e.g. acquisition parameters).

As primate researchers move toward open sharing of neuroimaging data, we urge them to share as much metadata as possible and to use the BIDS format, which is already accepted in PRIME-DE. Here, we have provided suggestions about what metadata are relevant, and where it can be shared in the BIDS format. These suggestions are not meant to be definitive and should be seen as a starting point for further discussion within the whole primate neuroimaging community. The community will need to come together to agree what, where, and how metadata should be shared precisely. Metadata sharing protocols could then be published on the PRIMatE-Resource Exchange website (https://prime-re.github.io/). In particular, a major challenge for primate open-science moving forward will be to develop standardized description of recommended metadata which can be shared in the form of common .*json* files (e.g. nomenclature for animal housing, common ethogram for spontaneous behaviour, formal naming convention for primate cognitive/behavioural tasks (though see Poldrak et al., 2011). We encourage NHP researchers to discuss and agree collectively how to incorporate metadata in the BIDS format, to ensure optimal progress in the field of NHP neuroimaging.

## Data and Code Availability Statement

Data were used for illustration purpose only.

## Declaration of Competing Interest

The authors declare no competing interests.

## Appendix Supplementary materials

Video 1 A male baboon performing the bimanual coordinated tube task Image, video 1

## Footnotes

Supplementary material associated with this article can be found, in the online version, at doi:10.1016/j.neuroimage.2020.117679.
